# FAS mediates apoptosis, inflammation, and treatment of pathogen infection

**DOI:** 10.3389/fcimb.2025.1561102

**Published:** 2025-04-22

**Authors:** Liying Hu, Juane Lu, Hongfei Fan, Changcheng Niu, Yanping Han, Qinggele Caiyin, Hao Wu, Jianjun Qiao

**Affiliations:** ^1^ School of Chemical Engineering and Technology, Tianjin University, Tianjin, China; ^2^ Zhejiang Research Institute of Tianjin University (Shaoxing), Shaoxing, China; ^3^ Tianjin Key Laboratory of Food Science and Biotechnology, College of Biotechnology and Food Science, Tianjin University of Commerce, Tianjin, China; ^4^ Key Laboratory of Systems Bioengineering (Ministry of Education), Tianjin University, Tianjin, China; ^5^ State Key Laboratory of Synthetic Biology, Tianjin University, Tianjin, China

**Keywords:** FAS, bacteria, virus, apoptosis, inflammation, treatment

## Abstract

The FAS cell surface death receptor, a member of the tumor necrosis factor receptor family, activates both apoptotic and non-apoptotic signaling upon interaction with its ligand FASL. It is critical in cell migration, invasion, immune responses, and carcinogenesis. Pathogen infection can influence host cells’ behavior by modulating the FAS/FASL pathway, thereby influencing disease progression. Understanding the role of FAS signaling in the context of pathogen interactions is therefore crucial. This review examines FAS-mediated apoptotic and non-apoptotic signaling pathways, with particular emphasis on the mechanisms of apoptosis and inflammation induced by bacterial and viral infections. Additionally, it highlights therapeutic strategies, including drug, cytokine, antibody, and FASL recombinant protein therapies, providing new directions for treating pathogenic infections and cancers, as well as insights into developing novel therapeutic approaches.

## 1 Introduction

FAS (also known as CD95, APO-1), a member of the tumor necrosis factor receptor family, is ubiquitously expressed on the surface of most cells and tissues, particularly on immune cells such as activated macrophages and T cells (Nagata and Golstein, 1995; Park et al., 2003). Discovered by Trauth et al., 1989, FAS was identified as a cell surface protein that induces apoptosis upon interaction with the APO-1 monoclonal antibody (Trauth et al., 1989). In 1991, Nagata’s group cloned this receptor protein. Two years later, they cloned its homologous ligand, FASL (CD95L), which is primarily expressed on activated T cells and natural killer (NK) cells. T cells trigger apoptosis and exert cytotoxic effects on target cells by binding FASL to FAS (Itoh et al., 1991; Suda et al., 1993; Kägi et al., 1994; Oshimi et al., 1996; Malyshkina et al., 2017).

The FAS protein has a molecular weight of approximately 48 kDa and is composed of three primary regions: an extracellular domain containing three cysteine-rich subdomains essential for homotrimerization, a transmembrane structural domain, and a cytoplasmic region that includes a death domain and a potential negative regulatory region (Behrmann et al., 1994; Consonni et al., 2022). Activation of FAS triggers apoptosis, thereby inhibiting abnormal cell proliferation. Dysfunctional FAS signaling, however, can lead to autoimmune diseases or contribute to cancer progression (Nagata and Golstein, 1995). Impaired or mutated FAS signaling can activate pro-survival pathways, such as NF-κB and MAPK/ERK, enhancing cancer cell proliferation, migration, and inflammatory microenvironment formation (O’Brien et al., 2005). In advanced cancers, tumor cells may evade cytotoxic T-cell killing by upregulating FASL and downregulating FAS, creating an immune-privileged microenvironment that drives malignancy (O’Brien et al., 2005). FAS participates in both apoptotic and non-apoptotic signaling pathways and plays a pivotal role in cell migration, invasion, immune regulation, and cancer development.

Pathogens, including bacteria and viruses, can modulate host cell FAS-mediated signaling pathways through various strategies to facilitate their survival and replication. For example, *Yersinia pestis* degrades cell surface FASL via its protease Pla, thereby inhibiting FAS-mediated apoptosis and suppressing the release of inflammatory cytokine. This reduces immune cell recruitment, creating an immunosuppressive microenvironment that facilitates bacterial survival and replication (Caulfield et al., 2014). The novel pandemic influenza A virus (H1N1) upregulates FASL to enhance the extrinsic apoptotic pathway. This regulation likely maintains viral replicative niches within host cells either by promoting viral release during cell death or by exploiting the apoptotic environment to suppress host immune clearance (Wang et al., 2014). Additionally, other pathogens including parasites (such as *Toxoplasma gondii*, and *Trypanosoma cruzi*) manipulate the FAS/FASL signaling pathway to facilitate infection (Nakajima-Shimada et al., 2000; Vutova et al., 2007). However, pathogen manipulation of the FAS/FASL signaling pathway to resist host immune responses triggers persistent inflammation, organ damage, and tumor metastasis, thereby exacerbating disease progression. Recent advances in research have elucidated both the macro-regulation and micro-molecular mechanisms of pathogen interactions with FAS-mediated pathways, offering new therapeutic opportunities. Current treatment strategies targeting FAS include chemotherapy, radiotherapy, cytokine therapy, monoclonal antibody therapy, etc., which are primarily applied to cancers and autoimmune diseases associated with FAS gene mutations (Risso et al., 2022). In addition, natural small-molecule drugs have emerged as promising treatments for FAS-related diseases in recent years. These novel approaches offer valuable insights into managing pathogen-induced FAS-mediated inflammation and associated disorders.

This review discusses FAS-mediated apoptotic and non-apoptotic signaling pathways, focusing on the mechanisms of FAS-mediated apoptosis and inflammation triggered by bacterial and viral infections. It also highlights recent advancements in therapies targeting the FAS/FASL system, providing valuable insights into the pathogen-induced disease mechanisms and the development of innovative therapeutic strategies.

## 2 FAS-mediated signaling pathways

### 2.1 Apoptotic signaling pathway

**Figure 1:**
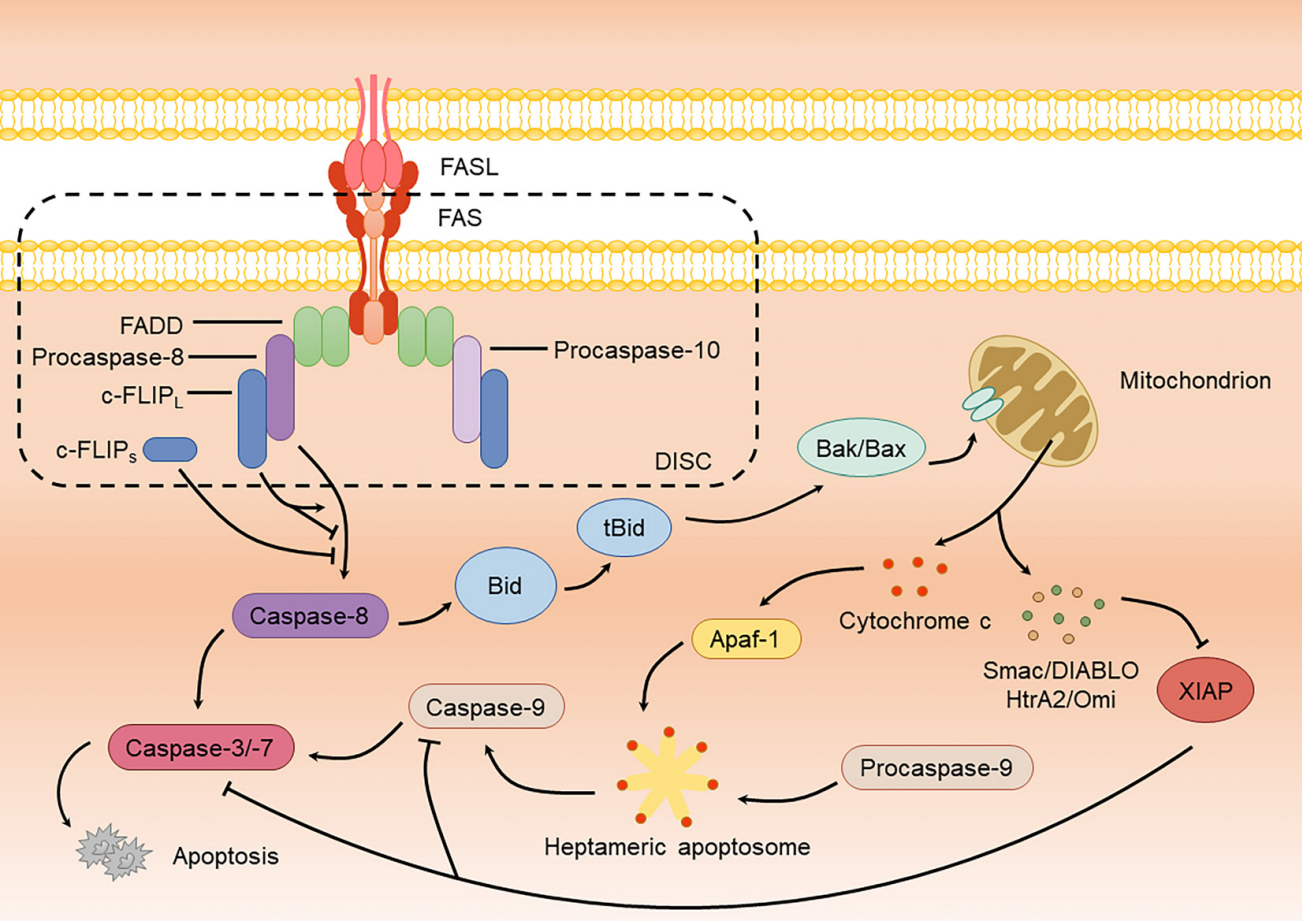
FAS-mediated apoptotic signaling pathway.

The FAS-mediated apoptotic signaling pathway has been extensively studied. FAS is activated by its homologous ligand, FASL, located on the plasma membrane, which promotes aggregation and triggers conformational changes. FASL, a cell-surface glycoprotein, exists in two forms: transmembrane (mFASL) and soluble (sFASL). While mFASL induces cell death, sFASL primarily activates non-apoptotic signaling pathways (O’Reilly et al., 2009; Risso et al., 2023). Upon binding to mFASL, FAS recruits the adaptor protein FAS-associated death domain (FADD), which recruits procaspase-8, procaspase-10, and cellular FLICE inhibitory protein (c-FLIP) via their homotypic death effector domain (DED), forming a membrane-bound death-inducing signaling complex (DISC) (Lavrik and Krammer, 2012; Ranjan and Pathak, 2024). Within the DISC, procaspase-8 is cleaved into its active form, which subsequently activates downstream caspases, such as caspase-3 and caspase-7, thereby initiating apoptosis (Lavrik and Krammer, 2012; Sahoo et al., 2023) (**Figure 1**). Notably, c-FLIP exists in two common isoforms: the long form (c-FLIP_L_) and short form (c-FLIP_S_), which regulate the FAS apoptotic pathway through interactions with procaspase-8. As a bifunctional regulator, c-FLIP_L_ can either promote or inhibit apoptosis. When binding to procaspase-8, it forms heterodimers that induce conformational changes, triggering partial autocleavage and allosteric activation of caspase-8 to initiate apoptosis (Chang et al., 2002; Yu et al., 2009). However, studies show that c-FLIP_L_ may also block complete caspase-8 processing by retaining cleaved caspase-8 within the DISC, thereby terminating apoptotic signaling (Micheau et al., 2002). Its controversial pro- or anti-apoptotic roles appear context-dependent, influenced by concentration and cell type (Krueger et al., 2001). In contrast, c-FLIP_S_ forms dysfunctional heterodimers with procaspase-8, and its truncated C-terminal domain fails to support procaspase-8 cleavage, thereby completely inhibiting DISC-mediated caspase-8 activation and apoptosis initiation (Schleich et al., 2016).

The aforementioned apoptotic pathway is categorized as extrinsic apoptosis with rapid DISC formation and significant caspase-8 production. In contrast, intrinsic apoptosis is more dependent on mitochondria and produces lower amounts of DISC and caspase-8 (Galluzzi et al., 2018). In this pathway, a small amount of caspase-8 cleaves Bid (BH3-interacting domain death agonist, belonging to the Bcl-2 family) (Kim et al., 2023). The truncated form of Bid (tBid) interacts with pro-apoptotic proteins Bak or Bax, also members of the Bcl-2 family, promoting their oligomerization and resulting in mitochondrial outer membrane permeabilization (MOMP), which releases cytochrome c (Flores-Romero et al., 2022). Once released into the cytoplasm, cytochrome c binds to Apaf-1 (apoptotic protease activating factor 1), forming a heptameric apoptosome. The apoptosome recruits caspase-9 monomers through their caspase recruitment domains (CARDs), catalyzing their activation (Li et al., 2017; Bock and Tait, 2020). Active caspase-9 subsequently activates downstream caspase-3 and caspase-7, inducing apoptosis (Srinivasula et al., 1998) (**Figure 1**). During this process, mitochondria also release Smac/DIABLO (diablo IAP-binding mitochondrial protein) and HtrA2/Omi (serine protease HTRA2), which block the inhibition of caspase-3/-7/-9 by XIAP (X-chromosome-linked inhibitor of apoptosis protein), thereby ensuring the efficient progression of apoptosis (Bock and Tait, 2020) (**Figure 1**).

In addition to caspase-mediated apoptosis, FAS can also mediate necroptosis via RIP kinase. Under FAS stimulation, RIPK1 is recruited to the DISC for activation, subsequently binding to RIPK3 via the RHIM (RIP homotypic interaction motif) to form the necrosome. RIPK3 then phosphorylates MLKL (mixed lineage kinase domain-like protein), inducing its oligomerization, membrane insertion, and disruption of membrane integrity, which triggers necroptosis (Holler et al., 2000; Sun et al., 2012; Cai et al., 2014; Liu et al., 2021).

### 2.2 Non-apoptotic signaling pathways

**Figure 2:**
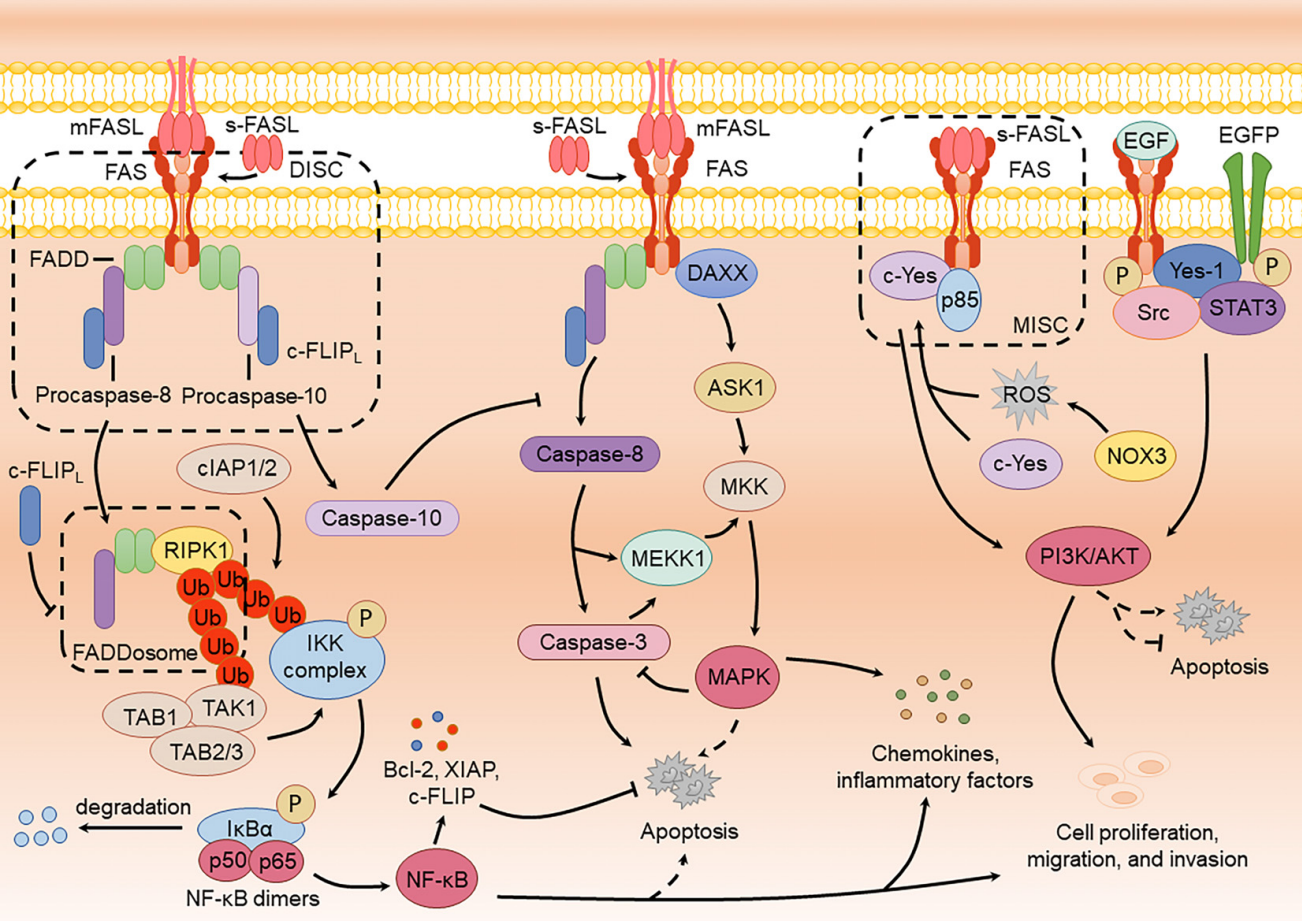
FAS-mediated non-apoptotic signaling pathways and the crosstalk with apoptotic signaling pathway.

Upon activation, FAS triggers signaling pathways such as NF-κB, MAPK, and PI3K/AKT through different mechanisms. These pathways regulate immune responses and promote cell proliferation, migration, and invasion by inducing the production of inflammatory factors and chemokines (**Figure 2**).

#### 2.2.1 NF-κB signaling pathway

Studies have shown that FAS activation can induce both apoptotic and NF-κB signaling within a single cell (Schmidt et al., 2015). However, the robust apoptotic response often obscures NF-κB activation. Upon FAS activation by FASL, the death domain recruits RIPK1 to FADD, forming a cytoplasmic procaspase-8-FADD-RIPK1 complex termed the “FADDosome” or “Complex II”. Within this complex, RIPK1 recruits the E3 ubiquitin ligases cIAP1/2 (cellular inhibitor of apoptosis protein 1/2), mediating its ubiquitination to generate ubiquitin chains. These chains recruit TAK1 (TGF-β-activated kinase 1) along with its adaptors, TAB1 and TAB2/3, as well as the IKK complex. Activated TAK1 phosphorylates and activates the IKK complex, which subsequently phosphorylates IκBα, leading to its degradation. This releases NF-κB dimers (such as p50/p65) for nuclear translocation, initiating the expression of pro-inflammatory cytokines such as IL-8 and driving inflammatory and immune regulatory responses (Kreuz et al., 2004; Ea et al., 2006; Henry and Martin, 2017). Activation of the NF-κB pathway through FAS can also lead to cell proliferation, migration, and invasion. For example, Zhang et al. showed that low-dose sFASL activates the FAS-mediated FADD-FLIP-TRAF-NF-κB pathway, resulting in elevated vascular endothelial growth factor (VEGF) levels and enhanced proliferation and migration of brain endothelial cells (Zhang et al., 2015). Notably, caspase-8 plays a crucial scaffold role in FADDosome, and its enzymatic activity is unrelated to the production of pro-inflammatory factors. The presence of enzymatic activity can cleave RIPK1, potentially explaining the masking of NF-κB activation (Kreuz et al., 2004; Henry and Martin, 2017).

Multiple factors are involved in regulating the activation of NF-κB in FAS, including caspase-10, TRAF2 (TNF receptor-associated factor 2), IAPs (inhibitor of apoptosis proteins), and A20 (deubiquitinating enzyme A20) (Davidovich et al., 2023). For instance, Horn et al. demonstrated that caspase-10 negatively regulates caspase-8-mediated cell death while facilitating NF-κB activation (Horn et al., 2017). In contrast, c-FLIP (mainly c-FLIP_L_) inhibits FADDosome formation, thereby suppressing FAS-induced NF-κB signaling and the production of inflammatory cytokines (Davidovich et al., 2023). Despite these insights, the precise mechanism underlying the formation of FADDosome remains to be fully elucidated. It is still unclear whether this process results from natural detachment from the receptor or is driven by other enzymatic activities that cleave the complex, necessitating further investigation.

#### 2.2.2 MAPK signaling pathway

FAS is dependent on caspase activity to participate in inducing the activation of the MAPK signaling pathway. Ligand- or agonistic antibody-induced FAS activation generates active caspase-8 or caspase-3, which cleaves mitogen-activated protein kinase 1 (MEKK1), resulting in active MEKK1 (Deak et al., 1998; Park and Baek, 2022). Active MEKK1 interacts with mitogen-activated protein kinase kinases (MKKs), such as MKK6 and MKK7, leading to the phosphorylation of ERK, JNK, and p38 (Toyoshima et al., 1997; Park and Baek, 2022). These kinases, in turn, induce the production of inflammatory factors, enabling cells to resist external stimuli and enhance survival. Matsumoto et al. found that caspase-8 inhibition in HEK293 cells eliminates FAS-mediated JNK activation, thereby suppressing AP-1 and IL-8 production (Matsumoto et al., 2007). Similarly, Kober et al. reported that c-FLIP inhibits the MAPK pathway by preventing caspase-8 processing, resulting in a decrease in FAS-mediated activation of ERK and p38 (Kober et al., 2011). Activating the MAPK pathway through FAS can also lead to cell proliferation, migration, and invasion. Li et al. reported that oral cancer cell stemness and lung metastasis benefit from FAS and ERK phosphorylation (Li et al., 2022).

Alternatively, MAPK activation can occur independently of caspases. For example, FAS stimulation in primary T lymphocytes can induce ERK and p38 pathways even when caspases are inhibited (Scheller et al., 2002). This caspase-independent activation likely involves FAS interacting with the receptor-associated protein DAXX (death-domain associated protein 6), which subsequently interacts with apoptosis signal-regulating kinase 1 (ASK1), leading to MKK phosphorylation to induce JNK activation (Ichijo et al., 1997; Pleinis et al., 2017; Bogolyubova and Bogolyubov, 2021). Recent studies suggest that DAXX promotes the proliferation of ovarian cancer ascites cells by activating the ERK pathway and directly binding to CEBP-β (Liu et al., 2018). However, whether FAS plays a direct role in this pathway remains unclear and warrants further investigation.

#### 2.2.3 PI3K/AKT signaling pathway

FAS-mediated PI3K/AKT signaling mainly promotes cell proliferation, migration, and invasion. During this process, sFASL binds to FAS, recruiting the Src family kinase c-Yes and the PI3K subunit p85 via NADPH oxidase 3 (NOX3) and ROS production, forming a motility-inducing signaling complex (MISC) that activates the PI3K/AKT pathway (Kleber et al., 2008; Tauzin et al., 2011; Akhiani and Martner, 2022). Activation of this pathway induces NF-κB activation and the cleavage of the extracellular matrix by MMPs (matrix metalloproteinases), including fibronectin, laminin, and type IV collagen, thereby enhancing cell migration and invasion. Furthermore, phosphorylated FAS interacts with epidermal growth factor receptor (EGFR) to form a complex comprising FAS, EGFR, Yes-1, Src, and STAT3 (signal transducer and activator of transcription 3) (Ta et al., 2018). Upon epidermal growth factor (EGF) stimulation, phosphorylated FAS accumulates in the nucleus, facilitating the nuclear localization of phosphorylated EGFR and STAT3. This process promotes cyclin D1 expression and STAT3-mediated activation of the AKT and MAPK pathways, thereby enhancing cell proliferation and migration (Ta et al., 2018; Seyrek and Lavrik, 2019).

### 2.3 Crosstalk between apoptotic and non-apoptotic pathways

FAS-mediated signaling pathways exhibit considerable crosstalk. FAS-mediated NF-κB activation induces anti-apoptotic proteins (including Bcl-2 family members, c-FLIP, XIAP) through its target genes, thereby suppressing FAS-driven apoptosis and establishing a negative feedback loop (Schram and Rothstein, 2003; Bernal-Mizrachi et al., 2006). Paradoxically, NF-κB can also promote apoptosis under specific contexts. As reported by Jennewein et al., NF-κB enhances DISC assembly to facilitate FAS-mediated apoptosis in glioblastoma cells (Jennewein et al., 2012). Additionally, MAPK activation suppresses FAS-mediated apoptosis by inhibiting caspase-8/-3 activity, while paradoxically, p38 MAPK has been reported to activate the intrinsic apoptotic pathway via FAS in CD8^+^ T cells (Holmström et al., 1999; Alvarado-Kristensson et al., 2004; Farley et al., 2006). The PI3K/AKT pathway also exhibits dual regulatory roles in FAS-mediated apoptosis (Lu et al., 2006; Liu et al., 2019b). These findings underscore the complexity of FAS signaling regulation, where pathway strength and outcomes depend on cell type and the surrounding microenvironment. Furthermore, inflammatory cytokines generated by non-apoptotic FAS signaling may reciprocally modulate apoptotic pathways, highlighting their therapeutic implications in FAS-driven diseases.

## 3 FAS-mediated apoptosis and inflammation induced by pathogen infection

Bacteria and viruses, two distinct classes of pathogens, differ significantly in infection mechanisms. Bacteria are prokaryotes with independent metabolic systems and reproductive capabilities. They invade hosts by secreting toxins to directly damage cells or injecting effector proteins via the type III secretion system to manipulate host cell signaling (Cossart and Sansonetti, 2004). In contrast, viruses are parasitic and rely on host cells for replication (König and Stertz, 2015). Hosts use apoptosis as a defense mechanism to limit infection and eliminate pathogens, while pathogens can exploit this process to enhance their spread. Bacteria like *Salmonella* induce apoptosis to escape host cells or evade immune clearance, whereas others such as *Mycobacterium tuberculosis* (*M. tuberculosis*) suppress apoptosis to prolong intracellular survival (Monack et al., 1996; Oddo et al., 1998). Similarly, many viruses inhibit apoptosis to sustain host cells for replication, as seen in human papilloma virus’s (HPV) E6 protein degrading p53, while some (such as adenoviruses) later trigger apoptosis to release progeny virions (Tollefson et al., 1996; Filippova et al., 2004). The differing reliance on apoptosis between two pathogen types stems from their survival strategies: bacteria depend on active dissemination, using apoptosis as an auxiliary mechanism to breach host barriers, while viruses require replication-release cycles, making apoptosis regulation critical to their life cycle. Host immunity plays a critical role in balancing the impact of infection, with chemotactic and inflammatory factors recruiting immune cells to eliminate infected cells via apoptosis, though excessive immune responses may occur. Here, we focus on FAS/FASL-mediated apoptosis or inflammation induced by bacterial and viral infections (**Table 1**).

**Table 1:** Apoptosis and inflammation triggered by pathogen infection through FAS signaling.

Category	Name	Cell types affected by infection	FAS-mediated apoptosis and inflammation	Modes of regulation	References
Bacteria	*L. monocytogenes*	T cells	Upregulate surface expression of FASL on T cells and promote apoptosis of target cells that express FAS.	Direct regulation	(Zenewicz et al., 2004)
		NK cells, macrophages	Upregulate FASL in NK cells, stimulate FAS receptors on the surface of macrophages, and induce the production of IL-18/IL-1β.	Immune response	(Uchiyama et al., 2013)
		Intestinal cells	Upregulate FAS and FASL and enhance IL-8 production via TLRs.	Immune response	(Fernandes et al., 2014)
		T cells, B cells	Upregulate FASL in CD4^+^ T cells and promote apoptosis of target cells (such as B cells) that express FAS.	Immune response	(Kotov et al., 2018)
		Inflammatory monocytes	Upregulates c-FLIP, inhibit caspase-8, and block FAS/FASL-mediated apoptosis.	Immune response	(Maudet et al., 2022)
	*M. tuberculosis*	Human macrophages	Downregulate FAS and inhibit apoptosis.	Direct regulation	(Oddo et al., 1998)
		Murine macrophages	Upregulate FASL, promote apoptosis of lymphocytes expressing FAS.	Direct regulation	(Mustafa et al., 1999)
		Human macrophages THP-1	Upregulate hsa-let-7b-5p microRNA, thereby inhibiting FAS transcription and suppressing apoptosis.	Direct regulation	(Tripathi et al., 2018)
		Macrophages derived from mouse bone marrow	Downregulate FAS expression, inhibit apoptosis.	Direct regulation	(Yao et al., 2017)
		B cells	Inhibit the expression of FASL, thereby suppressing other cells apoptosis.	Immune response	(Loxton and van Rensburg, 2021)
	*E. coli*	Monocyte U937	Upregulate FAS/FASL in monocyte U937 and promote apoptosis.	Direct regulation	(Wang et al., 2011a)
		Monocytes	Reduce mFASL expression of cord blood mononuclear cells to weaken apoptosis of other effector cells expressing FAS.	Direct regulation	(Gille et al., 2013; Dreschers et al., 2019)
		HeLa cells	Block DISC assembly and caspase-8 cleavage, inhibiting FAS/FASL-mediated cell apoptosis.	Direct regulation	(Pollock et al., 2017)
	*H. pylori*	Gastric epithelial cells, lamina propria lymphocytes	Upregulate FAS/FASL and promote apoptosis	Direct regulation and immune response	(Rudi et al., 1998)
		Gastric epithelial cells (AGS)	Upregulate FAS and promote apoptosis.	Direct regulation	(Jones et al., 1999)
		Human macrophages THP-1, Murine macrophage RAW 264.7	Upregulate FAS in human and mouse macrophages to promote cell apoptosis.	Direct regulation	(Alvi et al., 2011; Ansari et al., 2014)
	*P. aeruginosa*	Murine lung epithelial cells	Upregulate FAS and FASL, promoting cell apoptosis.	Direct regulation	(Grassmé et al., 2000)
		Human conjunctiva epithelial Chang cells	Upregulate FAS, mediate mitochondrial depolarization and JNK activation.	Direct regulation	(Jendrossek et al., 2001)
		HeLa cells	Aggregate membrane FAS, activate the FADD-Caspase to promote apoptosis.	Direct regulation	(Alaoui-El-Azher et al., 2006)
		Corneas polymorphonuclear leukocytes, murine macrophages	Downregulate the expression of local inflammatory cytokines and chemokines (TNF-α, IL-1β, etc.) mediated by FAS.	Direct regulation and immune response	(Zhou et al., 2010)
	*Shigella*	Lamina propria lymphocytes	Upregulate FAS and FASL, promoting apoptosis.	Immune response	(Raqib et al., 2002)
	*Salmonella*	Monocytes, NK cells	Upregulate FAS in NK cells and promote apoptosis.	Immune response	(Blanco et al., 2001)
	*S. aureus*	Monocytes	Upregulate FASL and promote its release, inducing apoptosis.	Immune response	(Baran et al., 2001)
Viruses	HPV	Human keratinocyte cell line HaCaT	Downregulate FAS and inhibit cell apoptosis.	Direct regulation	(Kabsch and Alonso, 2002)
		Human osteosarcoma U_2_OS cells	Degrade FADD and inhibit apoptosis mediated by FAS/FASL.	Direct regulation	(Filippova et al., 2004)
		Head and neck cancer cells, mouse oropharyngeal epithelial cell lines	Degrade FAS through MARCHF8 and inhibit apoptosis.	Direct regulation	(Khalil et al., 2023)
		Squamous cervical cancer cell lines	E2 protein interacts with c-FLIP to promote FAS-mediated cell apoptosis.	Direct regulation	(Wang et al., 2011b)
	HSV	Mouse epithelial Hepa 1-6 cells, mouse keratinocyte cell line 291.03C, neutrophils	FAS/FASL regulates apoptosis and inflammatory response within the vaginal mucosa, including the expression of CXCL1/2, TNF-α, and IL-1β.	Direct regulation and immune response	(Krzyzowska et al., 2011)
		Mouse monocyte RAW 264.7	Upregulate FAS and FASL in the early infection stage, promote apoptosis and CXCL9, TNF-α expression.	Direct regulation and immune response	(Krzyzowska et al., 2013)
		Microglia, astrocytes, monocytes, T cells, NK cells	FAS/FASL mediates HSV-1 brain infection and neuroinflammation.	Direct regulation and immune response	(Krzyzowska et al., 2021)
		Corneal cells, lymphocyte	Mediate lymphocyte apoptosis through the FAS/FASL pathway and inhibit keratitis.	Direct regulation and immune response	(Morris et al., 2012)
		Neonatal neutrophils	Enhance the expression of FAS and FASL, facilitating apoptosis in neonatal neutrophils.	Direct regulation	(Ennaciri et al., 2006)
	HIV	CD4^+^ T cells	Upregulate FASL and promote apoptosis of CD4^+^ T cells that express FAS.	Direct regulation	(Westendorp et al., 1995; Ghare et al., 2021)
		Jurkat cells	Pro-apoptotic proteins promote replication, while anti-apoptotic proteins inhibit replication, with opposite latency periods.	Direct regulation	(Wang et al., 2011c, 2022)
		Neutrophils	Activate FAS/FASL, upregulate FASL expression of neutrophils, and promote neighboring neutrophils apoptosis that expresses FAS.	Immune response	(Salmen et al., 2004)
		Macrophages, CD8^+^ T cells	Promote the expression of FASL on the surface of infected macrophages, bind with Fas on the surface of CD8^+^ T cells, and induce their apoptosis.	Direct regulation and immune response	(Mueller et al., 2001)
		Macrophages, T cells	Upregulate FASL in macrophages, promoting T cells apoptosis.	Direct regulation and immune response	(Dockrell et al., 1998)
		Renal tubular epithelial cells	Upregulate FAS and promote apoptosis.	Direct regulation	(Conaldi et al., 1998)
	HBV	Liver cells, T cells	From chronic hepatitis to cirrhosis, FAS/FASL is upregulated, and from cirrhosis to HCC, FAS/FASL is downregulated.	Direct regulation and immune response	(Mochizuki et al., 1996; Kondo et al., 1997; Bortolami et al., 2008)
		Liver cells	HBsAg promotes AKT-dependent FAS aggregation and procaspase-8 cleavage and promotes apoptosis.	Direct regulation	(Jing et al., 2018)
		Liver cells, mouse fibroblasts	HBx upregulates SAPK activity and inhibits FAS expression in liver cells through the SAPK/JNK pathway.	Direct regulation	(Diao et al., 2001)
		Liver cells, T cells	HBx upregulates Egr-2 and Egr-3, synergistically enhancing FASL expression in liver cells to kill T cells expressing FAS.	Direct regulation	(Yoo and Lee, 2004)
		Liver cells	Upregulate microRNA-181a, downregulate FAS, and inhibit apoptosis.	Direct regulation	(Zou et al., 2015)
		Liver cells	Activate JNK, downregulate antioxidant enzymes GPx and SOD2, make cells sensitive to the FAS pathway, but not induce cell apoptosis.	Direct regulation	(Wang et al., 2012)
		Liver cells	HBeAg (HBV e antigen) and HBc (HBV core protein) inhibit p53-dependent FAS expression, enhance sFAS (soluble FAS) expression, and suppress cell apoptosis.	Direct regulation	(Liu et al., 2015, 2019a)
		Liver cells	Activate PI3K/AKT and inhibit FAS-mediated apoptosis.	Direct regulation	(Wu et al., 2018)
		Renal tubular epithelial cell line: NRK-52E cells	Activate FAS/FASL through the MLK3-MKK7-JNK3 axis, mediating apoptosis of NRK-52E cells.	Direct regulation	(He et al., 2016)
	IV	HeLa cells	Upregulate the expression of FAS and FASL, promote apoptosis.	Direct regulation	(Takizawa et al., 1995; Fujimoto et al., 1998)
		Madin-Darby canine kidney (MDCK) cells, African green monkey kidney cells (Vero), A549 human lung carcinoma cells	Upregulate FAS and FASL, promote apoptosis.	Direct regulation	(Wurzer et al., 2004)
		Peripheral blood mononuclear leukocytes	Upregulate FAS on the surface of CD3^+^, CD4^+^, and CD8^+^ T cells, as well as FASL on the surface of monocytes-macrophages, promoting lymphocyte apoptosis.	Direct regulation and immune response	(Nichols et al., 2001)
		Dendritic cells, CD4^+^ T cells	Inhibit FASL expression through dendritic cells NLRC4, thereby suppressing apoptosis of T cells expressing FAS.	Immune response	(Hornick et al., 2019)
		Olfactory receptor neurons	Upregulate receptor neuron FASL, activate JNK pathway, and promote apoptosis of neighboring cells.	Direct regulation	(Mori et al., 2002)
	SARS-CoV-2	CD4^+^, CD8^+^ T cells	Upregulate FAS, sFASL, promote apoptosis.	Immune response	(Bellesi et al., 2020; André et al., 2022)
		Macrophages, airway epithelial cells	Directly upregulate FASL expression and IL-6 secretion in airway epithelial cells, upregulate FAS expression in macrophages, promote macrophage apoptosis and IL-1β secretion	Direct regulation and immune response	(Fraga-Silva et al., 2023)
		Inflammatory monocytic macrophages, NK cells, alveolar epithelial cells	Upregulate FASL in macrophages and NK cells, promote alveolar epithelial cells and immune cells apoptosis that express FAS.	Immune response	(Albert et al., 2024)
		CD8^+^ T cells, alveolar epithelial cells	CD8^+^ T cells expressing FASL bind to alveolar epithelial cells expressing FAS, inducing necroptosis of alveolar epithelial cells.	Immune response	(Komiya et al., 2024)
	EBV	Neutrophils	Upregulate FAS and mFASL, increase sFASL in culture, and promote cell apoptosis.	Direct regulation	(Larochelle et al., 1998)
		Lymphocytes	By regulating the expression of FASL in lymphocytes to induce T cell apoptosis and evade immunity.	Direct regulation and immune response	(Ohshima et al., 1999)
		T cells, B cells, macrophages	Upregulate FAS expression in T cells and FASL expression in B cells and macrophages, promoting T cell apoptosis.	Direct regulation and immune response	(Tanner and Alfieri, 1999)
		Intestine 407 cells	The encoded poly(A)-RNA releases protein kinase R (PKR) activity and inhibits FAS-mediated apoptosis.	Direct regulation	(Nanbo et al., 2005)
	DENV	Human umbilical vein endothelial cells	Upregulate the expression levels of FAS and FASL mRNA in human umbilical vein endothelial cells, promoting cell apoptosis.	Direct regulation	(Liao et al., 2010)
		Hepatocellular carcinoma cells HepG2	The interaction between capsid protein and DAXX promotes FAS-mediated apoptosis.	Direct regulation	(Limjindaporn et al., 2007; Netsawang et al., 2010)
	MV	Dendritic cells	Upregulate the expression of FAS in dendritic cells and induce apoptosis upon contact with activated T cells expressing FASL.	Direct regulation and immune response	(Servet-Delprat et al., 2000)

### 3.1 Bacteria

#### 3.1.1 Listeria monocytogenes

*Listeria monocytogenes* (*L. monocytogenes*), a foodborne pathogen, can cross the intestinal, blood-brain, and placental barriers, causing bacteremia and listeriosis (Kammoun et al., 2022). Early studies found that *L. monocytogenes* directly interacts with T cells through its virulence factors, listeriolysin O (LLO) and phosphatidylcholine-preferring phospholipase C (PC-PLC), promoting the surface expression of FASL. This FASL subsequently binds to FAS on neighboring cells, triggering apoptosis (Zenewicz et al., 2004). Later, Uchiyama et al. demonstrated that *L. monocytogenes* infection upregulates FASL expression in NK cells, which subsequently stimulates FAS receptors on the surface of macrophages, leading to IL-18/IL-1β production via a caspase-8 and ROS-dependent mechanism (Uchiyama et al., 2013). Fernandes et al. further reported that *L. monocytogenes* upregulates FAS, FASL and enhances IL-8 production via activating host Toll-like receptors (TLRs, TLR4 and TLR5) signaling (Fernandes et al., 2014). More recently, *L. monocytogenes* was shown to upregulate FASL expression in CD4^+^ T cells, inducing apoptosis in target cells that express FAS (Kotov et al., 2018). Additionally, the *L. monocytogenes* surface protein InlB upregulates c-FLIP through the c-Met-PI3Kα pathway, inhibiting caspase-8 activation and blocking FAS/FASL-mediated apoptosis, thereby facilitating bacterial invasion and spread (Maudet et al., 2022). These studies indicate that *L. monocytogenes* modulates FAS-mediated apoptosis and inflammatory responses through host immune mechanisms, rather than directly targeting FAS or FASL.

#### 3.1.2 Mycobacterium tuberculosis

*Mycobacterium tuberculosis* (*M. tuberculosis*), the causative agent of tuberculosis (TB), is primarily transmitted through airborne droplets and is highly infectious, often existing in a latent state (Palittapongarnpim et al., 2024). *M. tuberculosis* can bind to macrophages via TLRs and activate intracellular signaling pathways leading to macrophage apoptosis or necroptosis (Yao et al., 2017). However, it can also affect the survival of host cells through FAS. *M. tuberculosis* evades immune clearance by differentially modulating FAS/FASL in host macrophages. In human macrophages, *M. tuberculosis* downregulates FAS directly to inhibit apoptosis and promote cell survival, while in mouse macrophages, it upregulates FASL directly to kill FAS-expressing lymphocytes, protecting infected cells (Oddo et al., 1998; Mustafa et al., 1999). Tripathi et al. further demonstrated that *M. tuberculosis* suppresses FAS transcription in THP-1 human macrophages by upregulating hsa-let-7b-5p, which binds to the FAS 3’ untranslated region (3’UTR), thereby blocking apoptosis and enhancing bacterial persistence (Tripathi et al., 2018). These mechanisms underlie *M. tuberculosis*’s chronic infection strategy.

Clinical studies indicate that FASL expression in B cells of TB patients is significantly reduced compared to healthy individuals, but it returns to normal levels after six months of anti-tuberculosis treatment, suggesting its potential as a biomarker for treatment response (Loxton and van Rensburg, 2021). This indicates that *M. tuberculosis* suppresses the host’s immune response through the FAS/FASL system, thereby promoting the survival of infected cells. However, some studies have shown that apoptosis-related genes, such as FADD and caspase-8, are significantly upregulated in T cell subsets of TB patients, potentially driving T cells apoptosis through FAS-independent mechanisms (Canaday et al., 2001; Wang et al., 2023). Based on recent studies, *M. tuberculosis* infection inhibits the expression of FAS/FASL, thereby suppressing apoptosis. It also induces the expression of various inflammatory factors, but there is no direct evidence to suggest that these effects are mediated by FAS.

#### 3.1.3 Escherichia coli

*Escherichia coli* (*E. coli*), the most common gram-negative pathogen, is widely used as a model organism and is responsible for diseases such as colitis, diarrhea, and bacteremia (Gong et al., 2024). Studies investigating the interaction between *E. coli* and the FAS pathway primarily focus on monocytes as host cells. Wang et al. showed that *E. coli* infection directly upregulates FAS and FASL expression in U937 monocytes, triggering caspase-8-dependent apoptosis (Wang et al., 2011a). During bacterial infection, the apoptotic clearance of phagocytic monocytes suppresses the pro-inflammatory response in later stages (Dreschers et al., 2019). However, in neonatal monocytes, impaired apoptosis of phagocytic monocytes prolongs the pro-inflammatory phase, leading to persistent inflammation. Orlikowsky et al. showed that *E. coli* infection upregulates MMP-9 in cord blood mononuclear cells compared to peripheral blood mononuclear cells. MMP-9 cleaves mFASL, reducing cell surface FASL expression, which weakens apoptosis of FAS-expressing effector cells (such as T cells) and promotes persistent inflammation (Gille et al., 2013; Dreschers et al., 2019). Additionally, enteropathogenic *Escherichia coli* (EPEC) infection inhibits FAS-mediated apoptosis, primarily through the actions of the effector proteins NleB and NleF. *In vitro* infections, NleB glycosylates the death domain of FADD to block the formation of the DISC, thereby inhibiting caspase-8 activation. Conversely, NleF directly binds caspases-8, -4, and -9 to suppress their enzymatic activity (Pollock et al., 2017).

#### 3.1.4 Helicobacter pylori

*Helicobacter pylori* (*H. pylori*), a gram-negative bacterium transmitted via the fecal-oral route, is associated with chronic gastritis, peptic ulcers, and even gastric cancer (Engelsberger et al., 2024). Research on FAS-mediated apoptosis and inflammation caused by *H. pylori* infection is still in its early stages. Early studies show that *H. pylori* infection promotes apoptosis in gastric epithelial cells by directly upregulating FAS/FASL, or mediates cell clearance via host immune responses by upregulating FAS/FASL in lamina propria lymphocytes (Rudi et al., 1998). Other co-culture experiments with *H. pylori* and gastric epithelial cells have also shown increased FAS expression, which induces apoptosis (Jones et al., 1999). This upregulation may be linked to elevated levels of the p73 protein, *H. pylori* stabilizes the p73 protein via the cag pathogenicity island (cag PAI) and c-Abl tyrosine kinase, prolonging its half-life and directly inducing FAS transcription to promote apoptosis (Wei et al., 2008). Further studies have demonstrated that *H. pylori*-induced apoptosis predominantly occurs through the mitochondrial pathway. Additionally, *H. pylori* activates NF-κB, which exerts anti-apoptotic effects (Maeda et al., 2002). However, Hasumi et al. observed that *H. pylori* infection induces apoptosis even in cells lacking the FAS receptor, suggesting that while FAS enhances apoptosis, it is not the sole mechanism (Hasumi et al., 2002). Furthermore, *H. pylori* infection increases FAS expression in human and mouse macrophages, activating caspase-8 and inducing apoptosis, a process driven by the virulence factor HP986 (Alvi et al., 2011; Ansari et al., 2014).

#### 3.1.5 Pseudomonas aeruginosa

*Pseudomonas aeruginosa* (*P. aeruginosa*), a gram-negative bacterium, is associated with both acute and chronic infections, particularly in patients with cystic fibrosis (Jurado-Martín et al., 2021). Similar to *H. pylori*, research on the interaction between *P. aeruginosa* and the FAS pathway is still in its early stages. *P. aeruginosa* infection has been shown to directly upregulate FAS and FASL expression independently of the host immune response, thereby inducing apoptosis. The cystic fibrosis transmembrane conductance regulator (CFTR) also modulates FAS and FASL expression during infection (Grassmé et al., 2000; Cannon et al., 2003). Furthermore, Jendrossek et al. demonstrated that *P. aeruginosa* adheres to human conjunctiva epithelial Chang cells and directly upregulates FAS via its type III secretion system, leading to mitochondrial depolarization and JNK activation, thereby promoting apoptosis (Jendrossek et al., 2001). In addition, the ADP-ribosyltransferase activity of *P. aeruginosa* effector protein ExoS induces FAS receptor clustering in HeLa cells, activating FADD-caspase 8, but apoptosis remains unaffected by FASL-neutralizing antibody, suggesting alternative apoptotic mechanisms (Alaoui-El-Azher et al., 2006). *P. aeruginosa* infection also induces keratitis, a condition in which the regulation of the FAS/FASL pathway induces apoptosis and suppresses localized inflammatory expression of cytokines and chemokines, such as TNF-α and IL-1β. It also upregulates macrophage production of anti-inflammatory factor IL-10 to balance pro-inflammatory factor IL-12, demonstrating host immune responses against bacterial infection via the FAS/FASL system (Zhou et al., 2010).

#### 3.1.6 Other bacteria

Other bacterial pathogens, including *Shigella*, *Salmonella*, and *Staphylococcus aureus* (*S. aureus*), also influence apoptosis and inflammation through the FAS pathway (**Table 1**). *Shigella* infection induces upregulation of FAS and FASL in intestinal lamina propria lymphocytes via host immune responses, leading to apoptosis (Raqib et al., 2002). Blanco et al. showed that *Salmonella* strains, including wild-type Ty2 and mutant TYT1231, upregulate FAS expression in NK cells following monocyte infection and co-culture with NK cells, this may be due to the stimulation of cytokines produced after monocyte infection (Blanco et al., 2001). Furthermore, after being phagocytosed by monocytes, *S. aureus* induces FASL release on the monocyte surface, activating caspase-3 and initiating apoptosis in itself and neighboring cells via the FAS/FASL pathway (Baran et al., 2001).

### 3.2 Viruses

#### 3.2.1 Human papilloma virus

Human papilloma virus (HPV) is associated with several cancers, including cervical, head and neck, vaginal, and oropharyngeal cancer (Williamson, 2023). HPV is classified into low-risk and high-risk types, with high-risk types HPV16 and HPV18 receiving the most research attention (Williamson, 2023). The HPV genome encodes six early proteins (E1, E2, E4, E5, E6, E7) and two late proteins (L1, L2) (Nelson and Mirabello, 2023). Early studies indicate that the early proteins E5 and E6 play a critical role in inhibiting FAS-mediated apoptosis, thereby promoting HPV survival and replication within host cells (Nelson and Mirabello, 2023). The E5 protein, a membrane protein, suppresses the expression of the FAS receptor on the surface of the human keratinocyte cell line HaCaT, thereby preventing the binding of FASL to the receptor and inhibiting apoptosis (Kabsch and Alonso, 2002). In contrast, the E6 protein is widely recognized for its ability to rapidly ubiquitinate and degrade the tumor suppressor p53. Furthermore, E6 protects cells from FAS-mediated apoptosis by binding to the death domain of FADD and accelerating its degradation, which enhances viral survival and dissemination (Filippova et al., 2004). Recent studies have revealed that E6 upregulates the expression of the membrane-associated RING-CH-type finger ubiquitin ligase MARCHF8 via the MYC/MAX complex. MARCHF8 ubiquitinates FAS, leading to its degradation by the proteasome or lysosome and further inhibiting FAS-mediated apoptosis (Khalil et al., 2023). Additionally, the E2 protein interacts with c-FLIP, relieving the inhibition of c-FLIP on apoptosis signaling and promoting apoptosis. However, during HPV integration into the host genome, E2 protein expression is disrupted, impairing its anti-cancer effects (Wang et al., 2011b). In summary, HPV infection mainly promotes viral replication and spread in host cells by directly inhibiting the host cell FAS/FASL system and apoptosis.

#### 3.2.2 Herpes simplex virus

Herpes simplex virus (HSV), a neurotropic pathogen responsible for chronic human infections, contains two types: HSV-1 and HSV-2 (Rathbun and Szpara, 2021; Wijesinghe et al., 2021). It primarily causes skin and mucosal disorders, with a predilection for the oral and genital regions (Rathbun and Szpara, 2021; Wijesinghe et al., 2021). Krzyzowska et al. found that *in vitro* HSV-2 infection of murine epithelial and keratinocyte cells upregulated FAS, FASL, anti-apoptotic proteins (Bcl-2, NF-κB, and AKT kinase), and pro-inflammatory/chemotactic cytokines (CXCL1/2, TNF-α, and IL-1β). The early induction of anti-apoptotic proteins delayed cell death, resulting in only moderate apoptosis at later stages. Additionally, cytokines secreted by keratinocytes facilitated neutrophil migration toward infected cells, exacerbating inflammation (Krzyzowska et al., 2011). These findings highlight the role of the FAS pathway in regulating apoptosis and inflammatory responses during HSV-2 infection.

Additionally, HSV-2 infection of monocytes induced early upregulation of FAS and FASL, rendering them susceptible to FAS-stimulated apoptosis. FAS/FASL signaling further enhanced CXCL9 and TNF-α expression in cells, thereby recruiting other immune cells and potentiating local immune responses (Krzyzowska et al., 2013). Recent research has also revealed that the FAS/FASL pathway is involved in HSV-1-mediated brain infection and neuroinflammation. HSV-1 infection upregulated FAS and FASL expression in brain microglia and astrocytes, along with pro-inflammatory/chemotactic cytokines (such as CXCL9, CXCL10, TNF-α, IL-6), recruiting FASL-expressing immune cells (such as T cells) to clear infected cells (Krzyzowska et al., 2021). Interestingly, exogenous FAS stimulation on infected cells suppressed inflammatory mediator expression. This indicates that immune cell recruitment to infection sites disrupts FAS-mediated apoptosis and pro-inflammatory responses, with this paradoxical regulation exacerbating local inflammation (Krzyzowska et al., 2021). Moreover, the FAS/FASL pathway also plays a role in HSV-1-induced corneal inflammation and neonatal neutrophil apoptosis (Ennaciri et al., 2006; Morris et al., 2012) (**Table 1**).

#### 3.2.3 Human immunodeficiency virus

Human immunodeficiency virus (HIV) infection leads to acquired immunodeficiency syndrome (AIDS), severely impairing the immune system and increasing susceptibility to opportunistic infections and tumors (Kumah et al., 2023). A defining feature of HIV infection is the progressive depletion of CD4^+^ T cells, driven by multiple mechanisms including the FAS/FASL signaling pathway. Research indicates that exogenous FAS stimulation induces apoptosis in HIV-infected CD4^+^ T cells (Katsikis et al., 1995). The HIV-1 Tat and gp120 proteins upregulate FASL expression in T cells, which binds to FAS-expressing neighboring cells and the subsequent apoptosis of CD4^+^ T cells (Westendorp et al., 1995). Recent studies have elucidated the epigenetic mechanisms involved in this process. HIV-1 infection modifies histone H3 at the FASL promoter, increasing H3K4-trimethylation (H3K4Me3) and H3K9-acetylation (H3K9Ac) while reducing H3K9-trimethylation (H3K9Me3). These changes enhance FASL expression, making cells that express FAS more prone to apoptosis (Ghare et al., 2021). However, early studies also show that even with caspase inhibition or FAS deficiency, HIV-1 can still induce apoptosis *in vitro*, indicating that HIV-1 can directly kill T cells via FAS-independent pathways (Gandhi et al., 1998).

Wang et al. explored the effect of FAS/FASL-related apoptosis pathway molecules on HIV-1 replication. In Jurkat cells, the expression of pro-apoptotic proteins such as FASL, FADD, and p53 promoted viral replication, whereas anti-apoptotic factors like c-FLIP, Bcl-XL, and XIAP inhibited replication (Wang et al., 2011c). Notably, during HIV-1 latency, these proteins exhibited opposing effects, underscoring the intricate regulatory role of the FAS/FASL pathway in viral dynamics (Wang et al., 2022). HIV infection also increases the sensitivity of neutrophils, CD8^+^ T cells, macrophages, and renal tubular epithelial cells to FAS/FASL-mediated apoptosis, compounding the damage to the host’s immune and organ systems (Conaldi et al., 1998; Dockrell et al., 1998; Mueller et al., 2001; Salmen et al., 2004) (**Table 1**).

#### 3.2.4 Hepatitis B virus

Hepatitis B virus (HBV) is a hepatotropic virus infecting approximately 30 million individuals annually. Chronic HBV infection can progress to severe liver conditions, including cirrhosis and hepatocellular carcinoma (HCC) (Asandem et al., 2024). The FAS signaling pathway plays distinct roles at various stages of liver disease. From chronic hepatitis to cirrhosis, the expression of FAS and FASL increases, promoting apoptosis, whereas their levels decline during the transition from cirrhosis to HCC, reducing cell death and facilitating tumor progression (Mochizuki et al., 1996; Kondo et al., 1997; Bortolami et al., 2008).

During chronic hepatitis, HBV surface antigen (HBsAg) induces endoplasmic reticulum (ER) stress, which inactivates upstream AKT signaling molecules (PDPK1 and mTORC2). This disruption reduces AKT phosphorylation, relieving its inhibitory effect on FAS aggregation and caspase-8 cleavage, ultimately promoting apoptosis (Jing et al., 2018).

Conversely, in HCC, HBV employs distinct mechanisms to inhibit apoptosis, supporting tumor development. Multiple HBV proteins contribute to this process (**Table 1**). For instance, HBx protein upregulates SAPK (stress-activated protein kinase) activity, inhibits FAS expression via the SAPK/JNK pathway, and protects hepatocytes from death (Diao et al., 2001). Additionally, HBx potently activates Egr-2 (early growth response factor-2) and Egr-3 transcription, synergistically enhancing FASL expression in hepatocytes to kill FAS-expressing T cells and evade immunity (Yoo and Lee, 2004). HBV infection also induces the promoter activity of microRNA-181a, leading to its upregulation. This microRNA subsequently targets and suppresses FAS expression, thereby facilitating tumor growth (Zou et al., 2015). Beyond its effects on liver cells, HBV infection contributes to extrahepatic diseases, as listed in **Table 1**.

#### 3.2.5 Influenza virus

Influenza virus (IV) infects human and animal respiratory tracts, causing highly contagious seasonal epidemics (Liang, 2023). Early studies have shown that IV infection triggers low production of IFN-β while upregulating the serine/threonine kinase PKR, which is also activated by viral dsRNA. Activated PKR promotes FAS expression, which interacts with FASL on the surface of infected cells, leading to apoptosis (Takizawa et al., 1995; Fujimoto et al., 1998). Additionally, NF-κB promotes co-expression of both FAS and FASL on infected cell surfaces, a critical process for viral replication (Wurzer et al., 2004). Later, Nichols et al. demonstrated influenza A virus (IAV) infection increases FAS on CD3^+^/CD4^+^/CD8^+^ T cells and FASL on monocytes/macrophages in peripheral blood, driving lymphocyte apoptosis (Nichols et al., 2001). However, recent studies reveal that dendritic cell NLRC4 (an intracellular pattern recognition receptor) suppresses FASL expression, protecting FAS-expressing CD4^+^ T cells from apoptosis during IAV infection (Hornick et al., 2019). These findings provide valuable insights into the mechanisms underlying disease progression and T-cell immune responses.

#### 3.2.6 Severe acute respiratory syndrome coronavirus 2

Severe acute respiratory syndrome coronavirus 2 (SARS-CoV-2), a novel β coronavirus, is the causative agent of coronavirus disease 2019 (COVID-19). Its clinical manifestations include fever, cough, dyspnea, pneumonia, and, in severe cases, death (Mohamadian et al., 2021). Similar to HIV infection, severe COVID-19 exhibits lymphopenia, which is closely associated with the FAS/FASL-mediated pathway. Multiple studies have demonstrated that FAS expression on T cell surfaces is significantly upregulated in severe patients, accompanied by elevated serum sFASL levels and caspase-3 activation, leading to apoptosis of CD4^+^ and CD8^+^ T cells (Bellesi et al., 2020; André et al., 2022). As discussed by Leonardi et al., FAS activation may suppress FOXO1 (forkhead box protein O1) via AKT phosphorylation, inhibiting FOXP3 (forkhead box protein P3) expression and promoting T-cell differentiation into effector phenotypes. In the context of COVID-19, this leads to excessive T-cell activation, tissue infiltration, and apoptosis, contributing to immune dysregulation (Leonardi and Proenca, 2020).

The FAS/FASL system plays a dual role in COVID-19 pathogenesis. In mouse-adapted SARS-CoV-2 models, FASL upregulation on inflammatory monocytes/macrophages and NK cells triggers alveolar epithelial and immune cell apoptosis via FAS receptor activation, causing lung injury and acute respiratory distress syndrome (ARDS) (Albert et al., 2024). In severe COVID-19 patients, FASL-expressing CD8^+^ T cells induce alveolar epithelial necroptosis through FAS receptor engagement, exacerbating pulmonary damage (Komiya et al., 2024). Additionally, SARS-CoV-2-infected airway epithelial cells upregulate FASL, promoting FAS-mediated macrophage death while releasing IL-6, which stimulates the production of pro-inflammatory cytokines such as IL-1β (Fraga-Silva et al., 2023). These findings underscore the dual nature of FAS/FASL signaling, balancing antiviral defense with pathological hyperinflammation and tissue injury.

#### 3.2.7 Other viruses

Other viruses, such as Epstein-Barr virus (EBV), dengue virus (DENV), and measles virus (MV) also mediate apoptosis via the FAS/FASL pathway (**Table 1**). EBV infection increases FAS and mFASL expression in neutrophils, with sFASL also elevated in culture supernatants. Direct cell-cell FAS/FASL crosslinking promotes apoptosis (Larochelle et al., 1998). DENV infection increases the expression of FAS and FASL mRNA in human umbilical vein endothelial cells, driving apoptotic cell death (Liao et al., 2010). Similarly, MV infection upregulates FAS expression in dendritic cells (DCs). Co-culture of DCs with activated T cells expressing FASL induces DC apoptosis, facilitating viral release (Servet-Delprat et al., 2000).

## 4 Treatment methods targeting the FAS/FASL system

Dysregulation of the FAS/FASL system during pathogen infections drives chronic inflammation or autoimmune damage, whereas targeted intervention strategies against this pathway demonstrate therapeutic potential.

### 4.1 Drug therapy

Certain chemical agents exhibit significant regulatory effects on the FAS/FASL pathway. For instance, anesthetics like chloral hydrate demonstrate anti-inflammatory and anti-infective properties by upregulating FAS expression in macrophages., This activation induces Caspase-8/-3-dependent apoptosis, thereby eliminating these cells (Cai et al., 2016). As a result, chloral hydrate suppresses lipopolysaccharide (LPS)-induced pro-inflammatory cytokine release, highlighting its potential to mitigate infection-driven inflammation through FAS signaling (Cai et al., 2016). During *M. tuberculosis* infection, glutathione-boosted NK cells upregulate FASL expression to bind FAS on infected monocytes, triggering apoptosis and suppressing intracellular bacterial proliferation (Guerra et al., 2012). Furthermore, in *H. pylori*-infected mouse models, prolonged rebamipide treatment downregulates Fas/FASL expression in gastric mucosal epithelial cells, reducing apoptosis and alleviating chronic inflammation. Concurrently, it suppresses NF-κB-mediated pro-inflammatory cytokine release, delaying the progression of precancerous lesion (Hahm et al., 2003).

Compared to synthetic drugs, natural small-molecule drugs offer notable advantages, including reduced toxicity and fewer adverse effects, making them compelling candidates for treating FAS-related diseases. For example, In HPV infection, luteolin markedly upregulates the expression of FAS, FASL, and FADD in HeLa cells, activating the caspase-8/-3 cascade to initiate the extrinsic apoptotic pathway. Concurrently, it synergizes with the mitochondrial pathway to trigger intrinsic apoptosis, establishing a dual pro-apoptotic mechanism that restores host-mediated clearance of infected cells (Ham et al., 2014). In contrast, during EV71 (Enterovirus 71) infection, baicalin suppresses viral 3D polymerase expression to block early replication, while concurrently inhibiting FASL and caspase-3 levels, thereby attenuating extrinsic apoptotic pathway activation. This dual action protects host cells from apoptosis and reduces viral dissemination and release (Li et al., 2015). Furthermore, recent studies have shown that ginsenoside Rb1 exhibits therapeutic potential in acute lung injury caused by *S. aureus*. Infection with *S. aureus* upregulates death receptor-related genes such as FAS in lung cells, while ginsenoside Rb1 reverses these changes, reducing apoptosis and achieving therapeutic benefits (Shaukat et al., 2021).

Collectively, these studies highlight the multifaceted effects of FAS/FASL-targeting agents, including the modulation of immune-mediated pathogen clearance, inhibition of excessive inflammation and tissue damage, and regulation of apoptosis in infected host cells. However, optimizing drug concentration is crucial, and thorough *in vivo* efficacy and safety assessments are necessary for the development of broad-spectrum anti-pathogen therapies.

### 4.2 Cytokine therapy

Both apoptotic and non-apoptotic FAS-mediated signaling pathways are critically regulated by cytokines, offering potential therapeutic avenues for FAS-related diseases. For example, In chronic hepatitis C induced by viral infection, interferon (IFN) therapy initially induces transient elevation of serum soluble FAS (sFAS) and sFASL, followed by increased membrane-bound FASL expression (Yoneyama et al., 2002). This dynamic modulation may preferentially amplify functional apoptotic signaling of membrane-bound FASL, thereby optimizing the balance between infected cell clearance and tissue damage. Additionally, IFN therapy regulates pathogen-infected cell clearance by immune cells. IFN-α directly stimulates peripheral blood mononuclear cells, selectively upregulating FASL expression on NK cells, thereby enhancing their cytotoxicity against FAS-sensitive targets and promoting NK cell-mediated pathogen elimination (Kirou et al., 2000). Additionally, IFN-γ activates FASL expression in CD8^+^ T cells, promoting the clearance of HCV-infected hepatocytes via the Fas/FASL pathway (Jirillo et al., 2000).

Similarly, interleukins (ILs) modulate immune cell-mediated clearance of infected cells via the FAS/FASL pathway. In mouse models of respiratory syncytial virus (RSV) infection, localized IL-4 overexpression upregulates FASL on CD4^+^ and CD8^+^ T cells, enhancing their cytotoxicity against FAS-expressing virus-infected cells. However, this nonspecific killing may induce collateral apoptosis in healthy tissues and exacerbate immune pathology (Aung and Graham, 2000). Therefore, FAS/FASL-targeting strategies involving ILs and IFNs must be carefully controlled in space and time, depending on the disease stage and immune context. Exploring combination therapies may offer a way to balance the competing demands of immune clearance and tissue damage.

### 4.3 Antibody therapy

The therapeutic potential of FAS/FASL-targeting antibodies was first revealed in 1989 with the identification of the APO-1 monoclonal antibody (Trauth et al., 1989). While FAS or FASL antibodies are primarily employed in treating autoimmune diseases and cancer, they also show promise against pathogen infections by either activating apoptosis in infected cells (via FAS antibodies) or blocking immune cell apoptosis (via FASL antibodies). For example, anti-FAS monoclonal antibodies eliminate HIV-infected cells without enhancing viral replication, effectively suppressing HIV transmission (Kobayashi et al., 1990). Similarly, FASL antibody treatment in simian immunodeficiency virus (SIV)-infected primates inhibits FAS-mediated apoptosis, preserving T/B lymphocyte survival and maintaining immune responses (Salvato et al., 2007; Poonia et al., 2009). However, certain FAS antibodies (such as Jo2) cause severe liver toxicity, necessitating the development of next-generation FAS antibodies (such as HFE7A) (Ichikawa et al., 2000). Additionally, combined therapy with IFN-γ and FAS antibody CH11 synergistically enhances apoptosis in human cytomegalovirus-infected cells by suppressing viral replication and inducing apoptosis, demonstrating dual therapeutic efficacy (Chaudhuri et al., 1999).

### 4.4 FASL recombinant proteins therapy

Advancements in genetic engineering have facilitated the development of recombinant FASL (rFASL) as a critical tool for dissecting apoptosis mechanisms and exploring therapeutic applications. Multiple rFASL expression systems, including baculoviral, adenoviral (*AdFASL*), and *Pichia pastoris* platforms, have been established to produce rFASL for both *in vitro* and *in vivo* apoptosis models (Mariani et al., 1996; Tanaka et al., 1997; Okuyama et al., 1998). Mountz et al. utilized FASL-deficient (*gld/gld*) mice infected with murine cytomegalovirus (MCMV) to demonstrate that rFASL exacerbates chronic inflammation by sustaining activated T-cell infiltration post-viral clearance (Zhang et al., 2000). Intravenous infusion of FASL-expressing antigen-presenting cells (APC-*AdFASL*) selectively targeted splenic marginal zones, inducing activated T-cell apoptosis and significantly reducing inflammation in the lungs, liver, and kidneys (Zhang et al., 2000). In a parallel study, localized *AdFASL* delivery to salivary glands attenuated MCMV-driven immune hyperactivity and alleviated chronic sialadenitis (Fleck et al., 2001). Importantly, this localized approach minimized systemic FASL activation-associated hepatotoxicity, supporting its clinical safety profile.

## 5 Perspectives

FAS cell surface death receptor is pivotal in both apoptotic and non-apoptotic signaling pathways, contributing to processes such as cell migration, invasion, immune regulation, and cancer progression. Although the role of FAS signaling in cancer and autoimmune disorders resulting from FAS mutations has been extensively studied, its function in pathogen-induced infections remains poorly understood. Pathogens, including bacteria and viruses, can cause a wide range of diseases with significant mortality. These pathogens can manipulate host cell immunity and survival by modulating the FAS signaling pathway. Consequently, exploring the mechanisms underlying FAS signaling in the context of pathogen infections is essential for enhancing our understanding of disease pathologies and targeted therapeutic strategies.

This article explores the mechanisms of FAS-mediated apoptosis and inflammation triggered by bacterial and viral infections, while also highlighting therapeutic strategies targeting the FAS/FASL system. Despite these advancements, the regulatory mechanisms governing the FAS signaling pathway remain highly intricate. Does pathogen infection directly or indirectly regulate FAS expression? What factors contribute to this regulation? How does host immunity dynamically interact with these processes? These questions require further investigation to uncover the precise dynamics of FAS signaling. In addition, although various therapeutic approaches targeting the FAS/FASL system have been developed, including drug therapy, cytokine therapy, antibody therapy, and FASL recombinant proteins therapy, most of these methods are still in the laboratory research stage and rarely enter clinical research, which is a significant challenge for researchers. Looking ahead, there is a pressing need to clarify additional FAS-regulated disease pathways and to develop innovative therapeutic strategies targeting the FAS/FASL system, ultimately paving the way for improved clinical applications.

## Glossary

FAS: tumor necrosis factor receptor superfamily member 6
FASL: FAS ligand
NK: natural killer
NF-κB: nuclear factor kappa-B
MAPK: mitogen-activated protein kinase
ERK: extracellular regulated protein kinases
mFASL: transmembrane FASL
sFASL: soluble FASL
FADD: FAS-associated death domain
c-FLIP: cellular FLICE inhibitory protein
DED: death effector domain
DISC: death-inducing signaling complex
Bid: BH3-interacting domain death agonist
tBid: truncated form of Bid
Bak: Bcl-2 homologous antagonist
Bax: apoptosis regulator Bax
MOMP: mitochondrial outer membrane permeabilization
Apaf-1: apoptotic protease activating factor 1
CARDs: caspase recruitment domains
Smac/DIABLO: diablo IAP-binding mitochondrial protein
HtrA2/Omi: serine protease HTRA2
XIAP: X-chromosome-linked inhibitor of apoptosis protein
RIPK1: receptor-interacting serine/threonine-protein kinase 1
RIPK3: receptor-interacting serine/threonine-protein kinase 3
RHIM: RIP homotypic interaction motif
MLKL: mixed lineage kinase domain-like protein
PI3K/AKT: phosphatidylinositol-3-kinase/protein kinase B
cIAP1/2: cellular inhibitor of apoptosis protein 1/2
TAK1: TGF-β-activated kinase 1
TAB: TAK1 binding protein
VEGF: vascular endothelial growth factor
TRAF2: TNF receptor-associated factor 2
IAPs: inhibitor of apoptosis proteins
A20: deubiquitinating enzyme A20
MEKK1: mitogen-activated protein kinase 1
MKKs: mitogen-activated protein kinase kinases
JNK: c-Jun N-terminal kinase
DAXX: death-domain associated protein 6
ASK1: apoptosis signal-regulating kinase 1
NOX3: NADPH oxidase 3
MISC: motility-inducing signaling complex
MMP: matrix metalloproteinase
EGFR: epidermal growth factor receptor
STAT3: signal transducer and activator of transcription 3
EGF: epidermal growth factor
LLO: listeriolysin O
PC-PLC: phosphatidylcholine-preferring phospholipase C
TLRs: Toll-like receptors
TB: tuberculosis
3’UTR: 3’ untranslated region
EPEC: enteropathogenic *Escherichia coli*
cag PAI: cag pathogenicity island
CFTR: cystic fibrosis transmembrane conductance regulator
HPV: Human papilloma virus
MARCHF8: membrane-associated RING-CH-type finger ubiquitin ligase
HSV: Herpes simplex virus
CXCL: C-X-C motif chemokine
HIV: Human immunodeficiency virus
AIDS: acquired immunodeficiency syndrome
HBV: Hepatitis B virus
HCC: hepatocellular carcinoma
HBsAg: HBV surface antigen
ER: endoplasmic reticulum
HBx: HBV x protein
SAPK: stress-activated protein kinase
Egr: early growth response factor
IV: influenza virus
IAV: influenza A virus
SARS-CoV-2: severe acute respiratory syndrome coronavirus 2
COVID-19: coronavirus disease 2019
FOXO1: forkhead box protein O1
FOXP3: forkhead box protein P3
ARDS: acute respiratory distress syndrome
EBV: Epstein-Barr virus
DENV: dengue virus
MV: measles virus
LPS: lipopolysaccharide
RSV: respiratory syncytial virus
SIV: simian immunodeficiency virus
MCMV: murine cytomegalovirus
HBeAg: HBV e antigen
sFAS: soluble FAS
HBc: HBV core protein
IFN: interferon
IL: interleukin
